# Exploring protective factors in a high-risk subsample: the pivotal role of paternal support in preventing depression in a cohort of young adults

**DOI:** 10.47626/2237-6089-2024-0804

**Published:** 2025-03-27

**Authors:** Barbara Tietbohl-Santos, Bruno Braga Montezano, Taiane de Azevedo Cardoso, Thaíse Campos Mondin, Fernanda Pedrotti Moreira, Luciano Dias de Mattos Souza, Ricardo Azevedo da Silva, Flavio Kapczinski, Karen Jansen, Ives Cavalcante Passos

**Affiliations:** 1 Universidade Federal do Rio Grande do Sul Porto Alegre RS Brazil Programa de Pós-Graduação em Psiquiatria e Ciências do Comportamento, Universidade Federal do Rio Grande do Sul (UFRGS), Porto Alegre, RS, Brazil.; 2 Hospital de Clínicas de Porto Alegre Laboratório de Psiquiatria Molecular Porto Alegre RS Brazil Laboratório de Psiquiatria Molecular, Hospital de Clínicas de Porto Alegre, Porto Alegre, RS, Brazil.; 3 McMaster University Department of Psychiatry and Behavioural Neurosciences Hamilton ON Canada Department of Psychiatry and Behavioural Neurosciences, McMaster University, Hamilton, ON, Canada.; 4 UFRGS Alliance Research Group Porto Alegre RS Brazil Alliance Research Group, UFRGS, Porto Alegre, RS, Brazil.; 5 Deakin University School of Medicine Institute for Mental and Physical Health and Clinical Translation Victoria Australia Institute for Mental and Physical Health and Clinical Translation (IMPACT), School of Medicine, Deakin University, Victoria, Australia.; 6 Universidade Católica de Pelotas Pelotas RS Brazil Programa de Pós-Graduação em Saúde e Comportamento, Universidade Católica de Pelotas, Pelotas, RS, Brazil.

**Keywords:** Protective factors, major depressive disorder, at-risk population, cohort, social support, paternal support, resilience

## Abstract

**Objective:**

Major depressive disorder (MDD) is a global concern due to its widespread prevalence and morbidity. It is crucial to identify protective factors in high-risk individuals, including those with a familial predisposition, maltreatment history, and socioeconomic vulnerabilities.

**Methods:**

We assessed a high-risk subsample within a young adult population cohort (n = 791; mean age = 31.94 [standard deviation {SD} = 2.18]) across three waves, using multiple regression models to analyze higher education, feeling supported, spirituality, psychotherapy access, higher socioeconomic status, involvement in activities, cohabitation, and family unity in waves 1 and 2 and their association with MDD resilience at wave 3.

**Results:**

In the high-risk group, MDD incidence was 13.7% (n = 24). Paternal support had a protective effect on MDD incidence (odds ratio [OR] = 0.366; 95% confidence interval [95%CI] 0.137 to 0.955; p = 0.040) and suicide attempt risk (OR = 0.380; 95%CI 0.150 to 0.956; p = 0.038). Higher resilience scores were also protective (OR = 0.975; 95%CI 0.953 to 0.997; p = 0.030), correlating with reduced Beck Depression Inventory (BDI) (r = 0.0484; B = −0.2202; 95%CI −0.3572 to −0.0738; p = 0.003) and Montgomery-Åsberg Depression Rating Scale (MADRS) scores (r = 0.0485; B = −0.2204; 95%CI −0.3574 to −0.0741; p = 0.003).

**Conclusion:**

Our paper emphasizes reorienting the MDD approach, focusing on positive prevention strategies. It highlights the crucial role of fathers in family-based interventions and in promoting resilience in high-risk populations.

## Introduction

Major depressive disorder (MDD) has consistently occupied a prominent position among the leading 10 contributors to disability-adjusted life-years (DALYs) across diverse age brackets, retaining this status for individuals aged 10-49, as documented in the latest Global Burden of Diseases (GBD) report.^1^ Furthermore, presence of depression and anxiety in early life poses a significant threat to individual's future physical and mental well-being, educational achievements, financial stability, and interpersonal relationships.^2^ Given the far-reaching consequences of depressive episodes and the alarmingly high prevalence of this disorder, it is imperative to gain a deeper understanding of those individuals who are most susceptible to its development and the factors that either contribute to or mitigate its occurrence.

Parental mental health problems increase the risk of an individual experiencing a depressive episode by 42%^3^ and are a well-established risk factor for child psychopathology.^4^ Substantial research indicates that the offspring of depressed mothers are at increased risk for psychological and social maladjustment,^5^ and that children of depressed parents are more likely to experience depression, phobias, panic disorders, substance misuse, and problematic gaming during adolescence.^6,7^ It is worth noting that both genetic factors and the family environment make substantial and significant contributions to familial transmission of depression^8^ and other mental disorders.^9^

Protective factors can be viewed as positive traits and influences that can facilitate healthy development. Their significance does not necessarily lie in promotion of normal development in any environment, but they can play a crucial role when there is an interplay with risk factors.^10^ The most frequently discussed environmental factors encompass individual characteristics and various categories of supportive relationships, including parents, neighborhood, peers, and school.^11^ In addition, Askeland et al.^12^ associated individual factors such as goal orientation, self-confidence, social competence, social support, and family cohesion with a reduction in depressive symptoms. In contrast, Solmi et al.^13^ highlight the lack of convincing support for either risk or protective factors for MDD. Despite being extensively examined in cross-sectional research, these studies frequently lack the essential longitudinal dimension required for a comprehensive assessment of their impact on MDD prevention in high-risk individuals.^14^

Addressing this research gap, our study strives to enrich existing knowledge by providing a nuanced understanding of the effectiveness of various protective factors in preventing depressive symptoms among high-risk individuals. The primary objective is to leverage prior knowledge about protective factors and assess their preventative impact on depressive symptoms within this specific subsample of a population cohort comprising high-risk young adults.

## Methods

### Study design

This paper is a longitudinal study derived from a subsample of a population cohort. The first wave (T1) of data collection spanned from 2007 to 2009. The second wave (T2) occurred approximately 5 years later, spanning from 2012 to 2014, and the third wave (T3) was conducted from 2018 to 2020, roughly a decade after T1. All young adults who were part of the initial phase were invited to return for a follow-up assessment. Participants were informed about the research objectives and gave informed consent.

### Ethical considerations

The study was approved by the Research Ethics Committee at the Universidade Católica de Pelotas under protocol number 2008/118. Further information about the study design has been previously published elsewhere.^15^

### Participants

In the first wave, a total of 1,560 participants, aged 18 to 24 years, residing in urban Pelotas, state of Rio Grande do Sul, Brazil, were included. The rate of participation in the third follow-up assessments was 50.7%, with a total of 791 individuals (n = 791). The substantial loss to follow-up observed in our study is primarily attributable to disruptions caused by the coronavirus disease 2019 (COVID-19) pandemic. At T3, the average age of participants was 31.94 (standard deviation [SD] = 2.18) years. Respondents who were identified as having a psychiatric disorder were referred to appropriate healthcare services as required.

### Data sources/measurements

#### Sociodemographic characteristics

At T1, participants completed a comprehensive questionnaire covering various sociodemographic and economic items. These variables encompassed sex, skin color, age, marital status, years of education, occupational status, access to psychotherapy, and spirituality factors including participation in a religious group, attending religious services, and having a religion. In addition to these questions, participants were asked family-related questions regarding the structure of their family, such as cohabitation and the number of individuals within the family. Furthermore, individuals reported their economic classification according to Brazilian Association of Research Companies criteria (ABEP, Associação Brasileira de Empresas de Pesquisa).^16^

#### Social support

Perceived social support constitutes the respondent's subjective perception of the care and assistance received from social relationships. This perception encompasses emotional support (e.g., expressions of empathy), instrumental support (e.g., assistance with household tasks), and informational support (e.g., financial advice) that can be provided by various sources, such as friends or family.^17^ In our study, assessment of the subjective feeling of support was based on the responses to a series of dichotomous questions collected at T1. These questions covered whether respondents felt supported in general and specifically whether they felt supported by particular individuals within their family, including parents, siblings, partners, and their own children, if applicable.

#### Resilience

Resilience as a trait was measured using the Resilience Scale for Adults (RSA) at T2.^18^ The RSA consists of 33 items and employs a 7-point Likert response scale. It is designed to evaluate protective factors associated with personal attributes and support systems that have been demonstrated to promote adaptation in the face of psychosocial adversities.

#### Childhood Trauma Questionnaire

This retrospective, standardized, self-report instrument is specifically designed for assessing childhood trauma and is one of the most widely employed measures for this construct.^19^ The Childhood Trauma Questionnaire (CTQ) comprehensively examines five categories of maltreatment experiences – specifically, emotional abuse, physical abuse, sexual abuse, emotional neglect, and physical neglect – employing a Likert-scale approach to assess the severity of each incident. It is noteworthy that the instrument has undergone validation for use in Brazilian Portuguese.^20^ This questionnaire was also used to collect information about the participants’ sense of family unity during childhood.

#### High-risk for MDD

The high-risk criterion was determined by assessing participants’ family psychiatric history at T1, asking whether anyone in their family had ever been diagnosed with a psychiatric disorder. Participants who gave a positive response were then asked a series of questions related to each specific family member. To meet the criterion of high risk, at least one immediate family member needed to have a prior diagnosis of a mental health disorder. Notably, we initially explored the possibility of incorporating additional variables beyond participants’ family psychiatric history at T1 into our definition of high-risk for MDD. However, upon careful consideration, we found that including additional variables would result in a significantly restricted sample size for analysis.

#### Main outcome

Major depressive disorder was assessed by trained psychologists at each time point using the Mini International Neuropsychiatric Interview – PLUS (MINI-PLUS).^21^ In cases where there was uncertainty regarding the diagnosis of MDD, subjects underwent reassessment using the Structured Clinical Interview for Diagnostic and Statistical Manual of Mental Disorders (DSM)^22^ to confirm diagnosis.

#### Secondary outcomes

Furthermore, the MINI-PLUS administered at T3 was also utilized to gather clinical history information regarding depression severity, including the age of onset of first depressive disorder, history of inpatient psychiatric care, history of lifetime suicide attempts, and current suicide ideation. The severity of depressive symptoms was also evaluated at T3 using both the Montgomery-Åsberg Depression Rating Scale (MADRS)^23^ and the Beck Depression Inventory (BDI).^24^

### Variables

To assess demographic variables, we employed multinomial categorical variables for sex, skin color, age, marital status, and occupational status, along with economic classification based on the ABEP strata. Some variables were dichotomous, such as access to psychotherapy, participation in a religious group, attendance at religious services, having a religion, cohabitation with the individuals’ father and mother, perceived social support from those in the individuals’ social circle, as well as certain depression-related variables like previous inpatient psychiatric treatment, previous suicide attempts, and current suicidal ideation. Additionally, we generated quantitative variables to measure years of education, age at first depressive episode, resilience scores, and depression severity scores.

Creating a high-risk variable involved establishing a dichotomous measure for a positive immediate family history, after excluding individuals already diagnosed with MDD at baseline. Moreover, in relation to our main outcome – the absence of MDD at T3 – we established a dichotomous variable concerning the diagnosis of MDD according to the MINI-PLUS.

### Statistical methods

All statistical analyses were conducted using the R programming language (version 4.3.1), with the "tidyverse," "MASS," "dplyr," and "epiDisplay" packages. No imputation or adjustment for missing data were performed, i.e. the analysis was carried out exclusively on the observed cases. The number of individuals with missing data for each variable are shown in Supplementary Table S1. Significance was established at p < 0.05 in all statistical tests. The analysis was conducted in accordance with the following steps:

#### Group selection

Initially, participants were identified based on the high-risk criterion. This subsample was subsequently scrutinized with respect to our primary outcome – specifically, absence of MDD at T1 and presence of the diagnosis at T3. Following this, the cohort was stratified into four subgroups: "incident," "recurrent," "recovered," and "resilient." In this study, "incident" refers to individuals experiencing their first episode of depression at T3, "recurrent" denotes those with a history of depressive episode both at T1 and T3, "recovered" signifies individuals who have previously experienced depression at T1 but are asymptomatic at T3, and "resilient" characterizes participants who have never encountered depressive episodes despite being at risk. Given our focus on investigating protective factors in resilient individuals, we proceeded to compare the subgroup of high-risk individuals who experienced incident cases of MDD with those demonstrating resilience. This approach was taken as individuals classified in the recovered and recurrent groups could no longer be solely considered "at-risk" for depression.

#### Descriptive statistics and bivariate analyses

Initially, descriptive data were presented, detailing means and SDs, along with absolute and relative frequencies. Subsequently, we examined the incidence of MDD within the high-risk group and the entire sample. Following this, normality assessments were conducted for continuous variables using the Shapiro-Wilk test. The sociodemographic and economic characteristics of both groups were analyzed using the *t* test, chi-square test, or Mann-Whitney *U* test, as appropriate. The same methods were applied to assess the multiple proposed protective characteristics. Additionally, bivariate analyses explored group differences in relation to suicide attempts, current suicide risk, inpatient psychiatric treatment, and age at first depressive episode. Variables with a significance level of p < 0.200 in these analyses were included in the subsequent multivariate analyses.

#### Multivariate analyses

Logistic regressions were utilized to explore the connection between protective factors and resilience, examining group distinctions in relation to these factors and employing resilience to MDD (inverted incidence of MDD variable) as the dependent variable. Subsequently, logistic regressions were performed incorporating the protective factors previously identified as significant, now exploring various outcomes such as suicide attempts, current suicide risk, inpatient psychiatric treatment, and age at first depressive episode as dependent variables. This approach aimed to determine whether the protective factors identified as associated with resilience to MDD had implications for these crucial indicators of depression severity. Additionally, linear regressions were executed to delve into the associations between significant protective factors and the severity of MADRS and BDI depression scores. Finally, additional post-hoc bivariate analyses were conducted investigating differences between groups with higher and lower frequencies of the factors identified as protective and how these factors influenced various secondary measures of depression severity.

## Results

### Participants

At T3, complete data on depression incidence were available for 780 individuals. Subsequently, we excluded recurrent (n = 23) and recovered individuals (n = 66), focusing our analysis on the resilient (n = 627) and incident (n = 64) cases. These participants were then categorized based on our risk criteria into high-risk (n = 175) and normal-risk groups (n = 417). Observations with missing data for the risk criterion were omitted, resulting in a final participant count of 669 individuals. Among these, a significant difference in sex distribution between the groups was noted (p = 0.001), with females constituting 70.3% of the high-risk group (n = 123) and 55.9% of the normal-risk group (n = 233). No other significant differences were observed in sociodemographic variables, as detailed in Supplementary Table S2. At T3, the normal-risk group exhibited a 7.67% incidence of new MDD cases (n = 32). In contrast, the high-risk subgroup displayed an MDD incidence of 13.7% (n = 24), signifying a 78.5% higher incidence of depression compared to their normal-risk counterparts (p = 0.032).

Within the high-risk group (n = 175), no significant differences were observed in sociodemographic and economic characteristics between high-risk participants with and without a new diagnosis of MDD, as depicted in Table 1.

**Table 1:** Characteristics of the resilient to depression versus incident depression groups within the high-risk subsample

Characteristics		Resilient to depression high-risk group (n = 151)	Incident depression high-risk group (n = 24)	p-value
Gender*				0.0808
	Male	49 (32.5)	3 (12.5)	
	Female	102 (67.5)	21 (87.5)	
				
Age†		20.6 (1.92)	20.1 (2.05)	0.9532
				
	Skin color*			0.162
	Not white	44 (29.1)	11 (45.8)	
	White	107 (70.9)	13 (54.2)	
				
Economic classification*				0.0555
	High	84 (56.4)	8 (33.3)	
	Intermediate	61 (40.9)	16 (66.7)	
	Low	4 (2.7)	0 (0.0)	
				
Education*				0.261
	Incomplete high school or lower	59 (39.1)	15 (65.2)	
	High school	69 (45.7)	6 (26.1)	
	Secondary education	23 (15.2)	2 (8.7)	
				
Lives with father*				0.289
	Yes	65 (43.0)	7 (29.0)	
	No	86 (57.0)	17 (71.0)	
				
Divorced parents*				0.293
	Yes	96 (32.4)	11 (46.0)	
	No	170 (67.6)	13 (54.0)	
				
Paternal support*				0.00613
	Yes	104 (74.3)	10 (43.5)	
	No	36 (25.7)	13 (56.5)	
				
Maternal support*				0.283
	Yes	135 (92.5)	20 (83.3)	
	No	11 (7.5)	4 (16.6)
				
Resilience score (RSA)‡		140.0 (127.0-151.0)	127.0 (110.0-139.0)	0.009698
CTQ scores‡		11.5 (6.0-20.0)	18.5 (9.5-30.0)	0.03253
				
Suicide attempt*				0.00000177
	Yes	12 (7.9)	11 (45.8)	
	No	139 (92.1)	13 (54.2)
				
Suicide ideation*				0.0552
	Yes	10 (6.6)	5 (20.8)	
	No	141 (93.4)	19 (79.2)	
				
Paternal diagnosis*				0.757
	Yes	33 (21.9)	4 (16.7)	
	No	118 (78.1)	20 (83.3)	
				
Maternal diagnosis*				0.639
	Yes	83 (55.0)	15 (62.5)	
	No	68 (45.0)	9 (37.5)	

CTQ = Childhood Trauma Questionnaire; RSA = Resilience Scale for Adults.

* Absolute and relative (%) frequencies, p-value according to chi-square test.

† Mean (standard deviation), p-value according to *t* test.

‡ Median (25th-75th percentiles), p-value according to Mann-Whitney *U* test.

### Descriptive data

The primary factors significantly protective against the incidence of MDD within the high-risk group included having a supportive father and exhibiting higher resilience scores. The subsequent data pertain to our initial comparisons between the incident group and resilient high-risk groups, followed by post-hoc analysis investigating differences between groups with higher and lower frequencies of the factors identified as protective.

#### Comparisons between incident and resilient high-risk groups

Participants in the resilient group were more likely to report having a supportive father (n = 104; 74.3%) compared to the incident group (n = 10; 43.5%; p = 0.006). Interestingly, the same pattern did not emerge for maternal support, as a majority of our sample reported feeling supported by their mothers. Additionally, resilient individuals reported higher resilience scores (140 [127-151]) compared to the incident group (127 [110-139]; p = 0.009). The resilient group appeared to have lower exposure to trauma, reflected in lower CTQ scores (11.5 [6-20]), in comparison with the incident group (18.5 [9.5-30]; p = 0.032). Resilient individuals also had a lower frequency of suicide attempts (n = 12; 7.9%) than incident individuals (n = 11 [45.8%]; p < 0.001). The groups did not significantly differ regarding suicidal ideation at T3, parental marital status, cohabitation with the father, parental mental health diagnosis, or other socioeconomic variables. Additional details are provided in Table 1. Differences between groups with a significance level of p < 0.200 were incorporated into the subsequent multivariate analysis. These comprised socioeconomic level, skin color, paternal support, resilience scores, and CTQ scores.

#### Comparisons between high-risk individuals according to presence versus absence of paternal support

Individuals who reported having a supportive father exhibited lower depression severity scores (MADRS = 0 [0-6]; BDI = 6.5 [1-16]) compared to those reporting an absent father (MADRS = 4 [2-16]; p = 0.0002 and BDI =13 [8-26]; p = 0.001). The presence of a supportive father was also correlated with lower rates of inpatient psychiatric treatment (p = 0.0422). Interestingly, these groups did not exhibit differences in resilience scores, presence of paternal psychiatric diagnosis, or CTQ scores. Furthermore, no distinctions were observed in socioeconomic characteristics, as shown in Table 2.

**Table 2:** Characteristics of high-risk individuals according to presence versus absence of paternal support

Characteristics		Presence of paternal support (n = 114)	Absence of paternal support (n = 49)	p-value
Gender*				0.171
	Male	37.0 (78.70)	10.0 (21.30)	
	Female	77.0 (66.30)	39.0 (33.60)	
				
Depressive symptoms (MADRS score)^†^		0.0 (0.0-6.0)	4.0 (2.0-16.0)	0.0002
Depressive symptoms (BDI score)^†^		6.5 (1.0-16.0)	13.0 (8.0-26.0)	0.00197
Resilience scores (RSA)^†^		138.0 (125.0-149.0)	134.0 (119.0-146.0)	0.4266
Age at first depressive episode*		20.0 (5.35)	17.7 (4.92)	0.0642
				
Suicide attempt (lifetime)^‡^				0.0520
	Yes	11 (9.6)	11 (22.4)	
	No	103 (90.4)	38 (77.6)	
				
Suicidal ideation (current)^‡^				0.162
	Yes	107 (93.8)	3 (85.7)	
	No	7 (6.2)	46 (93.9)	
				
Psychiatric inpatient treatment^‡^				0.0422
	Yes	0 (0.0)	3 (6.1)	
	No	114 (100.0)	46 (93.9)	
				
Paternal psychiatric diagnosis^‡^				0.858
	Yes	24 (21.0)	9 (18.4)	
	No	90 (79.0)	40 (81.6)	
				
CTQ^†^		11.0 (6.0-20.0)	15.0 (8.0-27.0)	0.037

BDI = Beck Depression Inventory; CTQ = Childhood Trauma Questionnaire; MADRS = Montgomery-Åsberg Depression Rating Scale; RSA = Resilience Scale for Adults.

* Mean (standard deviation), p-value according to *t* test.

† Median (25th-75th percentiles), p-value according to Mann-Whitney *U* test.

‡ Absolute and relative (%) frequencies, p-value according to chi-square test.

#### Comparisons between high-risk individuals with higher and lower resilience scores

Participants were stratified for comparative analysis based on the 25th (Q1≤ 124) and 75th percentiles (Q4 ≥ 149) of their RSA scores. Those who scored higher were older at baseline (mean = 20.92; SD = 1.82) than those who scored lower (mean = 20.13; SD = 2.00; p = 0.03832). Individuals with higher resilience scores also exhibited lower depression severity scores (MADRS = 2 [0-6]; BDI = 5 [2-11]) than those who had lower resilience scores (MADRS = 5 [1.5-14.5]; p = 0.0001 and BDI = 12 [4.75-27.2]; p = 0.002). Additionally, individuals with higher resilience scores had a lower frequency of suicide attempts (n = 4; 7.8%) compared to those with lower scores (n = 11; 25%; p = 0.045). It is noteworthy that the groups did not differ concerning their history of past trauma. Additional information about group characteristics regarding resilience scores can be found in Table 3.

**Table 3:** Characteristics according to resilience levels (high versus low)

Characteristics		High resilience (n = 51)	Low resilience (n = 44)	p-value
Sex*				0.0517
	Male	21 (41.2)	9 (20.4)	
	Female	30 (58.8)	35 (79.6)	
				
Age at baseline†		20.92 (1.82)	20.13 (2.00)	0.03832
				
Paternal support*				0.215
	Present	34 (77.3)	27 (62.8)	
	Absent	10 (22.7)	16 (37.2)	
				
Depressive symptoms (MADRS score)‡		2.0 (0.0-6.0)	5.0 (1.5-14.5)	0.001223
Depressive symptoms (BDI score)‡		5.0 (2.0-11.0)	12.0 (4.75-27.2)	0.00265
Age at first depressive episode†		20.84 (6.22)	19.55 (5.35)	0.3184
				
Suicide attempt (lifetime)*				0.0450
	Yes	4 (7.8)	11 (25.0)	
	No	47 (92.2)	33 (75.0)	
				
Suicidal ideation (current)*				0.0545
	Yes	2 (3.9)	8 (18.2)	
	No	49 (96.1)	36 (81.8)	
				
Inpatient psychiatric treatment*				1
	Yes	1 (1.9)	1 (2.3)	
	No	50 (98.1)	45 (97.7)	
				
Paternal psychiatric diagnosis*				0.172
	Yes	10 (19.6)	15 (34.0)	
	No	41 (80.4)	29 (66.0)	
				
Maternal psychiatric diagnosis*				1
	Yes	28(54.9)	24 (54.5)	
	No	23 (45.1)	20 (45.5)	
				
CTQ‡		12.0 (5.0-23.5)	18.0 (9.75-30.0)	0.05397

BDI = Beck Depression Inventory; CTQ = Childhood Trauma Questionnaire; MADRS = Montgomery-Åsberg Depression Rating Scale; RSA = Resilience Scale for Adults.

Individuals grouped according to first and fourth quartiles of the distribution of RSA scores (Q1 ≤ 124; Q4 ≥ 149).

* Absolute and relative (%) frequencies, p-value according to chi-square test.

† Mean (standard deviation), p-value according to *t* test.

‡ Median (25th-75th percentiles), p-value according to Mann-Whitney *U* test.

### Outcome data

#### Supportive father

The presence of a supportive father at T1 reduced the likelihood of developing depression at T3 by 63% (odds ratio [OR] = 0.366; 95% confidence interval [95%CI] 0.137 to 0.955; p = 0.040). Also, having a supportive father reduced the risk of suicide attempt at T3 by 62% (OR = 0.380; 95%CI 0.150 to 0.956; p = 0.038)

#### Resilience scores

Higher resilience scores were associated with a minor, albeit significant, effect on MDD prevention in high-risk individuals (OR =0.975; 95%CI 0.953 to 0.997; p = 0.030). Furthermore, there was also a small but significant correlation between the resilience scores and depression severity at T3, according to both the BDI scores (r = 0.0484; B = −0.2202; 95%CI −0.3572 to −0.0738; p = 0.003) and the MADRS scores (r = 0.0485; B = −0.2204; 95%CI −0.3574 to −0.0741; p = 0.003).

#### Other protective factors

Several other potential protective factors, including having a religion, participating in a religious group, attending religious services, having access to psychotherapy, higher socioeconomic status, involvement in educational or professional activities, cohabitation with mother or father, a sense of family unity during childhood, and feeling supported by siblings, mother, and/or spouse, were not found to be statistically significant for MDD prevention, as indicated in Supplementary Table S3.

## Discussion

**Figure 1:**
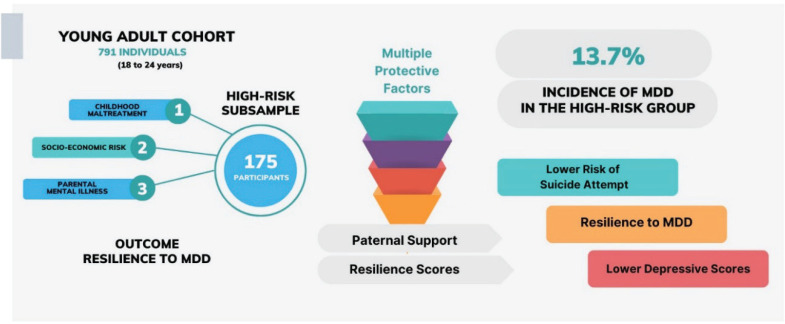
Comprehensive overview of study design and key findings.

This study delved into the influence of potential protective factors on the incidence of MDD within a subsample of a young adult cohort. As depicted in Figure 1, paternal support emerged as a critical factor, preventing MDD in high-risk individuals. This finding resonates with a recent meta-analysis that explored the dynamic nature of social support across the lifespan, underscoring the significance of parental support for adolescents, which evolves over time to encompass peer and spouse support.^25^ Furthermore, it aligns with the broader literature on social support, where cohort studies, meta-analyses, and systematic reviews have consistently demonstrated its protective effects against depressive symptoms, post-traumatic stress disorder (PTSD), and suicidal ideation in young adults.^25–29^ To our knowledge, this study is among the first to demonstrate how paternal support plays a significant protective role in averting the development of MDD in high-risk individuals in a large cohort of young adults.

Furthermore, it was revealed that having a supportive father not only decreases the severity of depressive symptoms, but also reduces the risk of suicide attempts. These findings resonate with other studies that have highlighted the protective effect of paternal support in the context of adolescent suicidality.^30^ Intriguingly, individuals who perceived support from their fathers did not demonstrate significant differences in resilience or trauma scores compared to those without such support in our study. Remarkably, even when controlling the analysis for these variables, paternal support still exhibited protective effects against MDD. This evidence implies that a supportive father may wield greater significance in MDD prevention than individual characteristics such as high resilience, even when considering past traumatic events. This phenomenon might be attributed to high-risk individuals, such as those with a positive family history of psychiatric disorders, potentially having lower intrinsic characteristics that contribute to better mental health outcomes, such as self-esteem^31^ and intelligence quotient (IQ).^32^ Consequently, they may rely more on positive influences received from their environment to prevent depression.

It is noteworthy that, contrary to expectations, while paternal support emerged as a significant factor for MDD prevention, maternal support did not. This contradicts previous findings highlighting the paramount influence of maternal support in averting MDD in children and adolescents.^33^ Given that a substantial majority (86.5%) of our overall sample reported feeling supported by their mothers, we hypothesize that the combined influence of positive maternal and paternal figures may be necessary to prevent MDD, as evidenced in previous studies.^34,35^ Indeed, it appears that the interaction of maternal and paternal parenting must be considered when predicting youth symptoms.^36^ Nevertheless, our study underscores the impactful role of a supportive father when maternal support is already in place.

Moreover, our study contributes to the body of literature by showing a small significant association between RSA scores and prevention of an MDD diagnosis, along with an inverse correlation between RSA scores and depressive symptoms scores. Extensive research has demonstrated that resilience plays a mediating role in the association between trauma and mood disorders^37,38^ and between victimization and suicidality^39^ and has been linked to overall better treatment outcomes for anxiety,^40^ PTSD,^26^ and even clinical illnesses.^41^ In fact, a recent meta-analysis demonstrated that individuals with mood disorders exhibit lower resilience compared to those without mood disorders.^42^ It is conceivable that more extensive studies with larger sample sizes may be requisite to comprehensively explore the nuanced aspects of resilience in relation to other MDD-related outcomes, such as the age at first depressive episode and the number of mood episodes, which did not attain significance in our analysis.

Our group's recent systematic review has highlighted several protective factors in high-risk cohorts, some of which could not be confirmed in the present study.^11^ Although other types of support, such as support from siblings, friends, and partners have been observed in multiple prior cross-sectional studies,^43–45^ they did not exhibit a significant protective effect in our study. Moreover, variables such as spirituality, access to psychiatric treatment/psychotherapy, engagement in educational activities, family composition, and family cohesion have previously demonstrated a protective effect on mental health outcomes.^46–50^ However, these factors did not exhibit a significant association with MDD prevention in our study. The complexities of these relationships and how they interact to shape resilience in high-risk circumstances warrant further investigation. Future studies are needed to better comprehend the intricate interplay of these factors.

While this study makes a valuable contribution to the literature, as there are few cohort studies that were able to assess how protective factors affect the incidence of MDD in high-risk individuals, it does have some limitations that should be considered. Firstly, the way the question was framed regarding support may introduce bias, since individuals can have a broad and subjective understanding of support. In addition, we did not analyze support in its various facets, such as emotional support or financial support. Additionally, the limited number of incident cases of MDD in high-risk individuals may have influenced the findings. The scarcity of male participants in the incident depressed group, with only three males, could introduce gender bias. Finally, the study did not inquire about the participants’ subjective feelings of support at T3, which means there is no evidence that the levels of perceived support remained consistent over time. These limitations should be taken into account when interpreting the results.

This young adult cohort study offers valuable insights into how a range of protective factors can influence the incidence of MDD in high-risk individuals. These findings have the potential to foster changes in the approach adopted in psychological interventions within this population. Rather than solely focusing on mitigating negative factors, the emphasis may shift towards actively promoting positive elements.^51^ Additionally, the study highlights the crucial role of engaging fathers and the significance of employing family-based strategies to enhance mental well-being in high-risk populations.
