# Supplementary material for: Exploring protective factors in a high-risk subsample: the pivotal role of paternal support in preventing depression in a cohort of young adults
Source: Trends Psychiatry Psychother. 2025 Mar 27;47:e20240804. doi: 10.47626/2237-6089-2024-0804 (PMC12677802; doi:10.47626/2237-6089-2024-0804)
Supplement: Supplementary file 1 [file 2238-0019-trends-47-e20240804-suppl01.pdf]

http://doi.org/10.47626/2237-6089-2024-0804

Supplementary Table S1 - Number of individuals without missing data for each variable used in the statistical analysis

Variable	Number of participants without missing data
Sex	1,560
Skin color	1,556
Age	1,548
Paternal support	1,422
Maternal support	1,474
Resilience scale total	1,239
Total MADRS Scale	779
Total BDI Scale	779
Having a religious belief	1,560
Belonging to a religious group	951
Sibling support	1,406
Friend support	1,434
Spouse support	1,209
Access to psychotherapy	212
Education	1,527
Socioeconomic level	1,238
CTQ scores	1,239
Presence of paternal diagnosis of mental disorder	624
Psychiatric inpatient treatment	779
Age at first depressive episode	248
Number of depressive episodes	176
Current suicidal ideation	779
Past suicide attempt	779

BDI = Beck Depression Inventory; CTQ = Childhood Trauma Questionnaire; MADRS = Montgomery-g Depression Rating Scale.

Supplementary Table S2 - Characteristics according to level of risk (total sample at T3)

Characteristics	Normal-risk group (n = 417)	High-risk group (n = 175)	p-value
Sex*			0.00149
Male	184 (44.1)	52 (29.7)	
Female	233 (55.9)	123 (70.3)	
Age at T3†	31.94 (2.21)	31.89 (2.07)	0.9532
Skin color*			0.506
Not white	118 (68.2)	55 (31.8)	
White	299 (71.4)	120 (28.6)	

* Absolute and relative (%) frequencies, p-value according to chi-square test.
† Mean (standard deviation), p-value according to *t* test.

Supplementary Table S3 - Statistical analysis of other potential protective factors

Protective factor	OR	95%CI	p-value
Maternal support	0.560	0.158 to 2.341	0.389
Sibling support	0.537	0.217 to 1.387	0.184
Friend support	0.690	0.281 to 1.768	0.423
Spouse support	2.445	0.765 to 9.634	0.157
Access to psychotherapy	3.790	0.048 to 1.950	0.276
Having a religious belief	1.091	0.946 to 1.235	0.186
Belonging to a religious group	1.382	0.357 to 4.489	0.607
Frequently attending to religious gatherings	0.840	0.544 to 1.270	0.416
Secondary education	0.510	0.227 to 1.047	0.081
Parents cohabitating (not separated)	0.734	0.308 to 1.779	0.485
CTQ scores	1.020	0.985 to 1.056	0.251

95%CI = 95% confidence interval; CTQ = Childhood Trauma Questionnaire; OR = odds ratio. Protective factors controlled for gender, ethnicity, and socioeconomic level, resilience scores, and CTQ scores. Factors proven not to be statistically significant.
